# Evaluating whole HIV-1 genome sequence for estimation of incidence and migration in a rural South African community

**DOI:** 10.12688/wellcomeopenres.17891.1

**Published:** 2022-06-21

**Authors:** Fabrícia F Nascimento, Manon Ragonnet-Cronin, Tanya Golubchik, Siva Danaviah, Anne Derache, Christophe Fraser, Erik Volz

**Affiliations:** 1 Department of Infectious Disease Epidemiology, Imperial College London, London, UK; 2 Big Data Institute, University of Oxford, Oxford, UK; 3 Africa Health Research Institute, Durban, South Africa

**Keywords:** HIV, phylodynamics

## Abstract

**Background:** South Africa has the largest number of people living with HIV (PLWHIV) in the world, with HIV prevalence and transmission patterns varying greatly between provinces. Transmission between regions is still poorly understood, but phylodynamics of HIV-1 evolution can reveal how many infections are attributable to contacts outside a given community. We analysed whole genome HIV-1 genetic sequences to estimate incidence and the proportion of transmissions between communities in Hlabisa, a rural South African community.

**Methods:** We separately analysed HIV-1 for *gag*, *pol*, and *env *genes sampled from 2,503 PLWHIV. We estimated time-scaled phylogenies by maximum likelihood under a molecular clock model. Phylodynamic models were fitted to time-scaled trees to estimate transmission rates, effective number of infections, incidence through time, and the proportion of infections imported to Hlabisa. We also partitioned time-scaled phylogenies with significantly different distributions of coalescent times.

**Results:** Phylodynamic analyses showed similar trends in epidemic growth rates between 1980 and 1990. Model-based estimates of incidence and effective number of infections were consistent across genes. Parameter estimates with *gag* were generally smaller than those estimated with *pol* and *env*. When estimating the proportions of new infections in Hlabisa from immigration or transmission from external sources, our posterior median estimates were 85% (95% credible interval (CI) = 78%–92%) for *gag*, 62% (CI = 40%–78%) for *pol*, and 77% (CI = 58%–90%) for *env *in 2015. Analysis of phylogenetic partitions by gene showed that most close global reference sequences clustered within a single partition. This suggests local evolving epidemics or potential unmeasured heterogeneity in the population.

**Conclusions:** We estimated consistent epidemic dynamic trends for *gag*, *pol *and *env *genes using phylodynamic models. There was a high probability that new infections were not attributable to endogenous transmission within Hlabisa, suggesting high inter-connectedness between communities in rural South Africa.

## Introduction

South Africa has the largest number of HIV-positive people in the world, with 7.5 million (95% CI = 6.9 million to 8.0 million) people living with HIV (PLWHIV) in 2019 ( Satoh & Boyer, 2019; UNAIDS, 2019). HIV prevalence varies between provinces with the lowest (8.3%) and highest (18.1%) prevalence observed in Northern Cape and KwaZulu-Natal provinces, respectively ( Simbayi *et al.*, 2019). HIV prevalence increased after 2012 corresponding to a period of increased uptake of antiretroviral therapy (ART) and reduced AIDS-related mortality ( Simbayi *et al.*, 2019).

The HIV epidemic in South Africa is dominated by HIV-1 subtype C ( Hemelaar *et al.*, 2011), which likely originated in Africa around 1960 (95% confidence interval (CI) = 1956 – 1964) ( Wilkinson *et al.*, 2015). Subtype C shows high genetic diversity in South Africa, as a result of multiple introductions from Zambia, Botswana, Malawi and Zimbabwe that occurred between 1985 and 2000, during a period of socio-political transformations in the country ( Wilkinson *et al.*, 2016). More recently, phylogenetic analyses of HIV-1 genetic sequences from South Africa and other African countries (Angola, Botswana, Democratic Republic of the Congo, Malawi, Mozambique, Swaziland, Tanzania, Zambia, and Zimbabwe) showed that as of 2014, 35% (95% CI = 20% – 60%) of new infections were attributable to external introductions into South Africa ( Rasmussen *et al.*, 2018). However, both studies ( Rasmussen *et al.*, 2018; Wilkinson *et al.*, 2016) included only sequences from within Africa to analyse external introductions into South Africa.

In this study, we evaluated HIV-1 subtype C genetic sequences from individuals from Hlabisa, a rural South African community in KwaZulu-Natal province. We evaluated the potential of whole genome HIV-1 sequence data and phylodynamic analysis methods ( Volz *et al.*, 2013) to inform estimates of recent epidemic trends in Hlabisa.

The objectives and design of this analysis were aligned with the previous analysis by Rasmussen *et al.* (2018), which was based on data from a neighbouring community in KwaZulu-Natal. Specifically, our aim was to evaluate the potential of randomly sampled sequence data to estimate (1) the probability that a new infection arises from endogenous transmission within South Africa versus importation from outside of South Africa, and (2) trends in recent HIV incidence.

## Methods

### Ethics approval

Ethical approval for the cohorts we used in this study were obtained from the institutional review board (IRB) from the University of Cape Town, Research Ethics Committee (IRB doc number 213/400), University of Witwatersrand, HREC (IRB doc number 40206 and 060809), and University of KwaZulu-Natal Biomedical Research Ethics Committee (UKZN BREC) (numbers BFC104/11, BF052-010, and BF110/09). All participants provided written informed consent.

### Sources of data

The data used in this study comprised HIV-1 genomic sequence data and metadata from 2,503 PLWHIV sampled in the Hlabisa sub-district of KwaZulu-Natal, South Africa. Samples came from five distinct cohorts, with most samples originating from the South African treatment as prevention trial (ZA-TasP) ( Derache *et al.*, 2019; Iwuji *et al.*, 2018; Rossouw *et al.*, 2017). Genetic sequencing data were generated by the PANGEA (Phylogenetics And Networks for Generalized Epidemics in Africa) consortium ( Abeler-Dörner *et al.*, 2019; Pillay *et al.*, 2015). Metadata included the sample dates, age and gender of participants, as well as CD4 cell counts. CD4 counts were utilised to determine the stage of HIV infection for each participant; however, most CD4 data were not collected concurrently with samples used for HIV sequencing which reduced the utility of these data. From the 2,503 PLWHIV that we analysed, 48% had a CD4 cell count carried out within six months of sequence data, while 12% had a CD4 count carried out more than six months after genetic sequencing. For the remaining 40%, no CD4 count was available.

It is common for phylodynamic papers to report results based solely on the *pol* gene as *pol* sequences are abundant in sequence databases ( Rasmussen *et al.*, 2018; Wilkinson *et al.*, 2019). We split genomic HIV-1 sequence data into *gag*, *pol*, and *env* genes using a subtype C reference sequence (GenBank accession number: KU319528) and the BLAST command line application tools version 2.5.0+. We chose to analyse the genomic data separated into genes 1) to reduce bias from recombination; 2) to have an appropriately calibrated substitution model and molecular clock model for each gene; and 3) to check whether phylodynamic analyses carried out using different genes yielded comparable results.

We used COMET ( Struck *et al.*, 2014) to determine the HIV-1 subtype of each genetic sequence. COMET classified less than 1% of sequences as non-subtype C and those were excluded from our analyses. Sequences with fewer than 800 nucleotides (nt) were also excluded from the analyses.

Because the global reservoir of HIV infections dwarfs the size of the sampled South African data, an imported lineage will have ancestral relationships that are much more strongly influenced by global HIV dynamics as opposed to local epidemiological dynamics. We accounted for this by identifying HIV sequences closely related to those within our collected South Africa data and including these sequences in our data set. We refer to these sequences as close global references (CGRs).

We were unable to select sequences from within South Africa outside of Hlabisa, as this information was not available in the sequence database. To select CGRs, we generated a global BLAST ( McGinnis & Madden, 2004) database consisting of all available HIV-1 sequences where location of sampling was known and did not correspond to South Africa. This database consisted of all matching sequences in GenBank combined with the PANGEA-generated sequences not yet available in GenBank. Countries included in PANGEA are South Africa, Botswana, Zambia, Uganda, Kenya and Tanzania. Our CGR database included 786,571 sequences, around 9,000 of which were from other PANGEA sites. For each South African sequence, we used the BLAST tool and custom Python scripts (see *Extended data* [ Nascimento, 2022]) to select the top three closest matches within the global database. Hypervariable regions of envelope ( *env*) gene sequences were manually removed prior to using BLAST to prevent spurious matches for the *env* gene. We removed CGRs which were labelled as non-functional provirus, non-functional or truncated genes, and hypermutated sequences, and we verified the subtype classification for each of these CGR sequences using COMET. Any CGR which was not classified as subtype C was removed. Note that multiple South African sequences can share a closest match within the global set, and overlapping BLAST matches were deduplicated. After deduplication and curation, 30%, 17%, and 35% of sequences in the alignment were CGRs for the *gag*, *pol*, and *env* genes, respectively.

***Sequence alignment, drug resistance sites and recombination.*** We used Gene Cutter to generate, for each gene, a combined genetic sequence codon alignment consisting of the sampled South African sequences and the top three deduplicated BLAST matches from the global set. In all alignments, we also included two sequences (accessions AY371157 and K03454) from subtype D to be used as an outgroup in phylogenetic analyses (see section on molecular clock analysis). We further removed an average of 36% of columns of the alignment in which the majority of sequences was missing a nucleotide ( Yang, 2014). The majority of these 36% of columns comprised nucleotide insertions observed in a single sequence and composed by unknown nucleotide (Ns).

Because drug resistance nucleotide sites are under strong selective pressure and may influence phylogenetic reconstruction, we next identified and masked drug resistance sites in the *pol* gene using the *big.phylo* R package version 1.0.0 ( Ratmann, 2018) and HXB2 (accession K03455) reference sequence. The *big.phylo* R package uses the drug resistance mutation sites from the International Antiviral Society-USA ( Wensing *et al.*, 2015).

We examined the *gag*, *pol* and *env* alignments for evidence of within-sample recombination using RDP4 ( Martin *et al.*, 2015). We used the default setting and the option “auto mask for optimal recombination detection” to detect recombination within each alignment. Three, four and nine sequences were identified as potential recombinants in *gag*, *pol* and *env* alignments, respectively, and these were excluded from subsequent analysis.

After data preparation, maximum likelihood trees were estimated using alignments consisting of 2,455 sequences for the *gag* (of which 730 sequences were CGRs); 2,821 for the *pol* gene (of which 482 sequences were CGRs); and 2,422 for the *env* gene (of which 840 sequences were CGRs) including South African, CGRs and outgroup sequences.

### Phylogenetic analysis

We estimated phylogenetic trees by maximum likelihood (ML) using RAxML-NG version 0.8.1 ( Kozlov *et al.*, 2019), the HKY + Γ (Hasegawa-Kishino-Yano ( Hasegawa *et al.*, 1985) plus gamma distribution ( Yang, 1996)) DNA substitution model, and using two partitions for the data (1+2 and 3rd codon positions). This model choice was motivated by previous findings that partitioning by codon position is often superior for protein-coding sequences ( Shapiro *et al.*, 2006).

To estimate the ML tree, we replicated phylogeny inference 50 times using random starting conditions. From these 50 fits, 25 used parsimony-based randomized stepwise addition trees as starting trees, and 25 used random trees as starting trees to search for the best ML tree. Similarly, to calculate branch support for each tree, we ran 100 independent bootstrapped trees per alignment in parallel which were merged into a single file to calculate the bootstrap support for each branch of the best ML tree.

***Molecular clock analysis.*** We estimated time-scaled phylogenetic trees using the ML trees using the uncorrelated lognormal relaxed clock model from the *treedater* R package version 1.0 ( Volz & Frost, 2017) and dates of sampling where known, taking into account the alignment lengths of each gene. For CGRs, a range of dates was provided based on year of sampling where available. A total of 12, six and 44 samples for *gag*, *pol* and *env*, respectively, were missing date of collection, and for these we provided *treedater* with a very broad range of sample time limits (1980 – 2015). Molecular clock outliers were identified and excluded using the method from Benjamini and Hochberg at a 5% level ( Benjamini & Hochberg, 1995), and *treedater* was re-run without outliers. Root position was based on a non-subtype C outgroup (accessions AY371157 and K03454). We also removed redundant CGR sequences from the trees. Redundant CGRs were sequences whose neighbour in the phylogenetic tree was also a CGR. Thus, the closest neighbour for a CGR was a sequence from South Africa.

After removing outliers and redundant CGRs, our final alignments used in phylodynamic analyses consisted of 1,962 sequences for the *gag* (of which 244 sequences were CGRs); 2,526 for the *pol* gene (of which 205 sequences were CGRs); and 1,766 for the *env* gene (of which 192 sequences were CGRs) including South African, CGRs and outgroup sequences.

**Figure 1.**
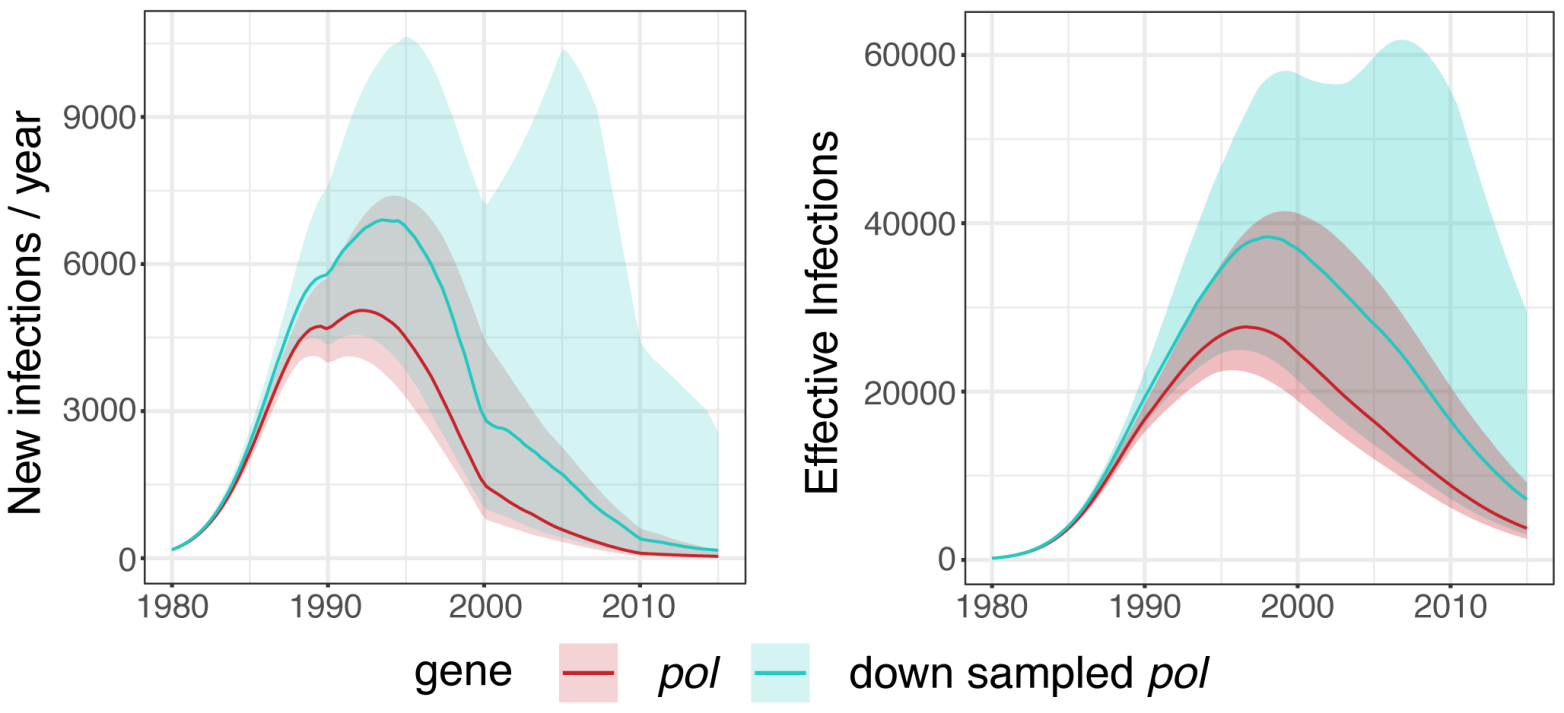
Epidemic dynamics in the South African data set for *pol* gene using *phydynR*. Left: New infections per year. Right: Effective number of infections per year. Posterior medians and 95% credible intervals are shown with solid line and shaded area, respectively.

We observed the highest number of sequences for the *pol* gene compared to *gag* and *env* genes. Because of that we down sampled the number of sequences in the *pol* alignment to those IDs observed in the *env* alignment. Our final alignment used in the phylodynamic analyses with *phydynR* (see section on phylodynamic analysis) for the down sampled *pol* gene consisted of 1,660 sequences (of which 194 were CGRs). Estimates of new infections and effective number of infections per year showed larger credible interval using the down sampled version of the *pol* alignment ( Figure 1). Further reports were based on the complete data for the *pol* alignment.

***Phylogenetic partitions.*** We used the *treestructure* method to partition time-scaled trees into clades with significantly different distributions of coalescent times ( Volz *et al.*, 2020). Phylogenetic partitions are correlated with metadata and provide insights into epidemiological structure, such as the presence or absence of association with geographic variables ( Volz *et al.*, 2020). We partitioned the South African data into smaller sets of two-three partitions with *n* ∈ (400,1500) for each gene. For *gag* and *pol*, *treestructure* identified three partitions, while for *env*, *treestructure* identified two partitions.

### Epidemiological models

We developed a mathematical model for epidemiological dynamics in Hlabisa, which was applied within a phylodynamic analysis framework to compute the likelihood of our genetic and clinical data. Our model was defined in terms of the number *I*( *t*) of infected and infectious HIV individuals, and the number *Z*( *t*) of infected individuals on ART as a function of time *t*. These variables described epidemic dynamics within the geographic region of focus, where sequence data were sampled. Additionally, we accounted for migration of HIV lineages between South Africa and the much larger global reservoir of HIV infections, denoted *X*( *t*). We used a simple two-parameter exponential function to describe the size of the global reservoir, which grows at rate *ρ*.

The rate of new infections was controlled by the function *β*( *t*) which specified the per-capita rate of transmissions from untreated individuals. The function *β*( *t*) was specified by a linear interpolating spline with slope changes in 1980, 1990, 2000, 2005, 2010, and 2015. This model avoids making strong assumptions about how incidence scales with prevalence and does not require modelling the number of susceptible individuals through time. The function *α*( *t*) specified the per-capita rate of initiating antiretroviral therapy. The model did not account for treatment failure or loss to follow up. The function *α*( *t*) was given by a simple one-parameter linear function which increased from zero starting in 2005. The parameters *µ* and *γ* denote per-capita rates of natural mortality and rates of disease-related mortality, respectively.

The dynamics of the number of infected individuals in each compartment is specified by the following system of ordinary differential equations where the time derivative of a variable *x* with respect to time is $\dot{x}$ :


$$\begin{array}{l} \dot{I}(t)=(\beta (t)-\mu -\gamma -\alpha (t))I(t)\\ \;\dot{Z}(t)=\alpha (t)I(t)-\mu Z(t)\\ \;\dot{X}(t)=\rho X(t) \end{array}\;(1)$$

### Phylodynamic analyses

We carried out fixed-tree phylodynamic analyses using dated phylogenies estimated by ML and *treedater*. This analysis was carried out with the *BayesianTools* R package version 0.1.6 ( Hartig *et al.*, 2018) which implements Bayesian Markov chain Monte Carlo (MCMC) methods and the *phydynR* R package version 0.2.0 ( Volz, 2017) which implements the structured coalescent likelihood.

The structured coalescent model made use of the PL1 approximation ( Volz & Siveroni, 2018) to the likelihood, which is slower to compute but more accurate. We estimated the parameters of our mathematical model (represented in Equations 1) using the differential-evolution MCMC zs sampler (MCMC-DEzs) ( ter Braak & Vrugt, 2008) as implemented in the *BayesianTools* R package. We initially ran few MCMC-DEzs for 18,000 iterations, and we chose one run per analyses to provide initial conditions for subsequent longer runs. We then run final MCMC-DEzs ranging from 24,000 to 29,000 iterations using different initial conditions depending on the analyses (for *gag*, *pol* and *env*). These MCMC-DEzs were run in parallel using the computing resources of Imperial College London. We merged five independent runs in order to have two sets of runs to compare posterior distributions for each parameter and assess convergence of the chains. We also used the Gelman diagnostics from *BayesianTools* to check for convergence. For a list of the parameters we estimated and their corresponding priors see Table 1.

**Table 1.** List of priors used in the Bayesian MCMC analyses.

Definition and symbol	Prior
Growth rate of global reservoir ( *ρ*)	Lognormal(-3.352, 1)
Importation rate as imports per infected per unit time	Lognormal(-2.9957, 1/4)
Initial number of global reservoir lineages ( *X*_0_)	Lognormal(9.9035, 0.25)
Initial numbers of infected and infectious HIV individuals ( *I*_0_)	Exponential(1/10)
Changes in the transmission rate in intervals of 10 or 5 years (1980, 1990, 2000, 2005, 2010 and 2015) ( *β*)	Lognormal(-1.792, 1)
Disease-related mortality rate ( *γ*)	Fixed at 1/10.2 ^a^
Natural mortality rate ( *µ*)	Fixed at 1/(62.7-30) ^b^

^a^ Based on Mangal (2017) who estimated the median survival time in Africa as 10.2 years when considering individuals 30 years old at seroconversion. ^b ^We used a life expectancy of 62.7 years for females in 2016 ( Life expectancy and Health Life expectancy Data by WHO region, WHO)

### Non-parametric coalescent analyses

We carried out a non-parametric coalescent analysis in order to estimate effective population size through time. This was carried out on fixed time-scaled phylogenies reconstructed from *gag*, *pol*, and *env* genes separately to establish congruence of phylodynamic results based on different genes. Estimates were based on partitioned time-scaled trees per gene estimated using *treedater* and *treestructure* as previously described. Prior to analysis, we pruned all CGR sequences from phylogenetic trees. These analyses were carried out with the *skygrowth* R package version 0.2.0 ( Volz & Didelot, 2018) using MCMC and default parameter settings.

## Results

Based on our epidemiological model, we were able to estimate the effective number of infections and the proportion of imports from outside Hlabisa as described below.

### Epidemic dynamics

**Figure 2.**
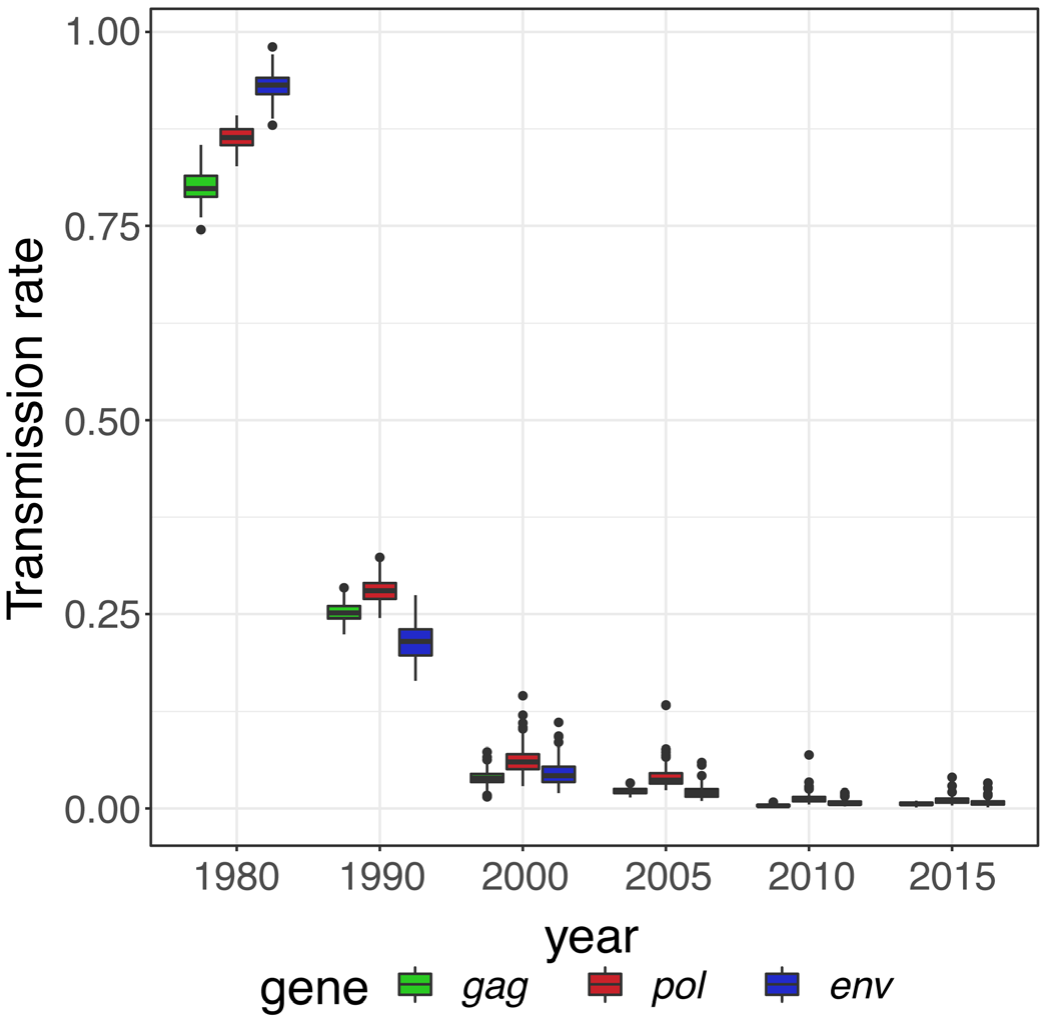
Transmission rates in the South Africa data set for *gag*, *pol* and *env* genes using *phydynR*. Boxplot showing the distribution of transmission rates per year.

**Figure 3.**
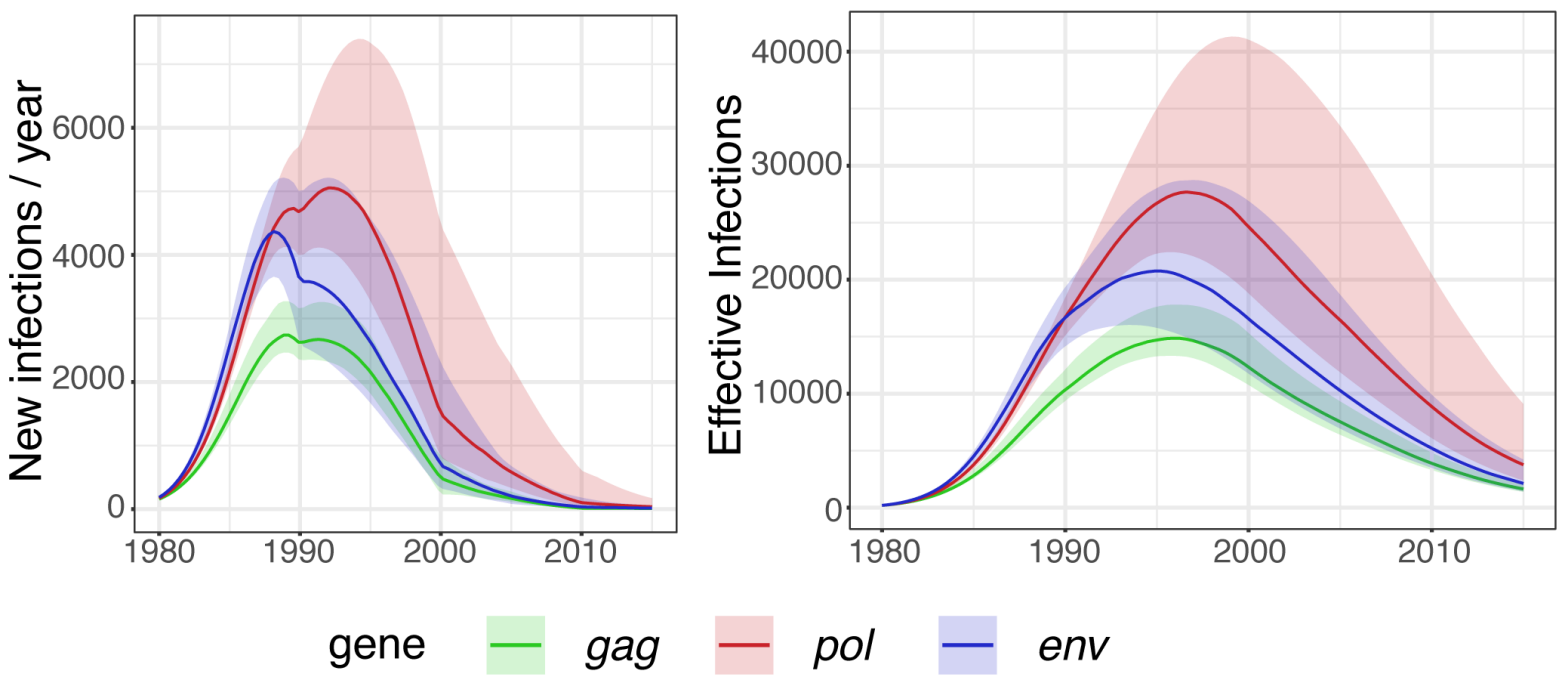
Epidemic dynamics in the South Africa data set for *gag*, *pol* and *env* genes using *phydynR*. Left: New infections per year. Right: Effective number of infections per year. Posterior medians and 95% credible intervals are shown with solid line and shaded area, respectively.

Coalescent analyses demonstrated rapid expansion of the epidemic during the 1980s and a smooth monotonic decrease of transmission rates into the 1990s ( Figure 2 and Figure 3). We observed similar transmission rate estimates for different genes, and the degree of similarity increased towards the present ( Figure 2).

Model-based estimates of new infections per year and of the effective number of infections over time based on different genes showed congruent trajectories ( Figure 3). Early estimates using the *gag* gene were lower than estimates using *pol* and *env* genes. The latter two were in agreement. From 1990, estimates using *gag*, *pol* and *env* genes were in agreement with overlapping credible intervals ( Figure 3). We also observed that estimates using the *pol* gene were higher than those estimated using the *gag* or *env* genes after 1990 ( Figure 3).

**Figure 4.**
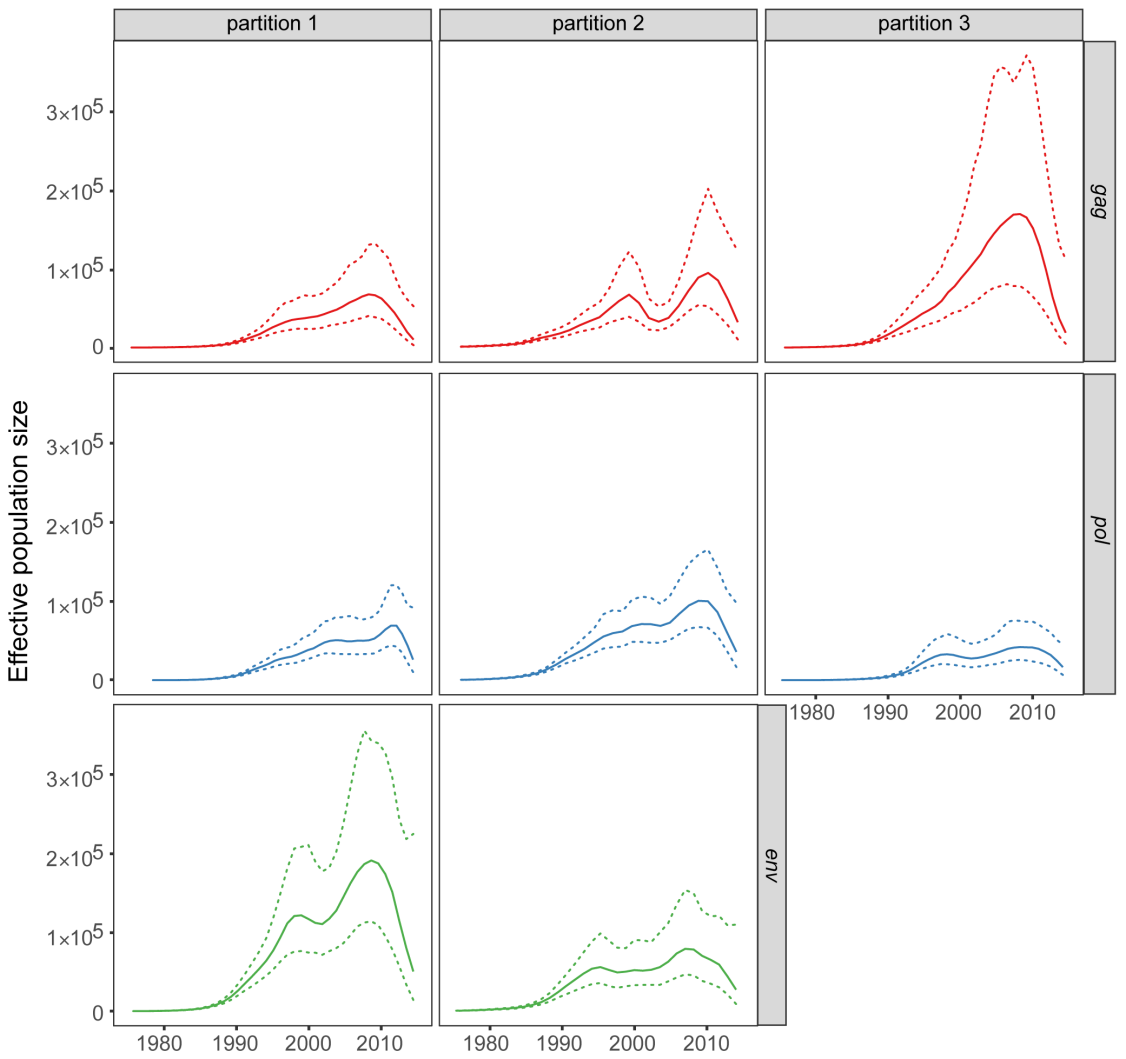
Effective population size through time using *skygrowth* based on time scaled phylogenetic partitions for *gag*, *pol* and *env* genes. Posterior medians and 95% credible intervals are shown with solid and dotted lines, respectively.

Non-parametric coalescent analysis to estimate the effective population size by gene and for each tree partition similarly showed agreement with the exception of *gag* partition 3 and *env* partition 1. These showed higher estimates and credible intervals than the other gene/partitions ( Figure 4). We also observed a sharp decrease in effective population size after 2010. In general, analysis of the *pol* gene across the different partitions showed a smaller credible interval than the *gag* and *env* genes ( Figure 4). This could be a consequence of the larger sample size for *pol* than for *gag* and *env*.

### Migration and the role of the global HIV reservoir

Finally, we estimated the probability that a new infection was not attributable to endogenous transmission in Hlabisa. Posterior median estimates for the proportion of transmissions from outside of Hlabisa were 85% (95% CI = 78% – 92%) for *gag*, 62% (CI = 40% – 78%) for *pol*, and 77% (CI = 58% – 90%) for *env* gene in 2015.

**Figure 5.**
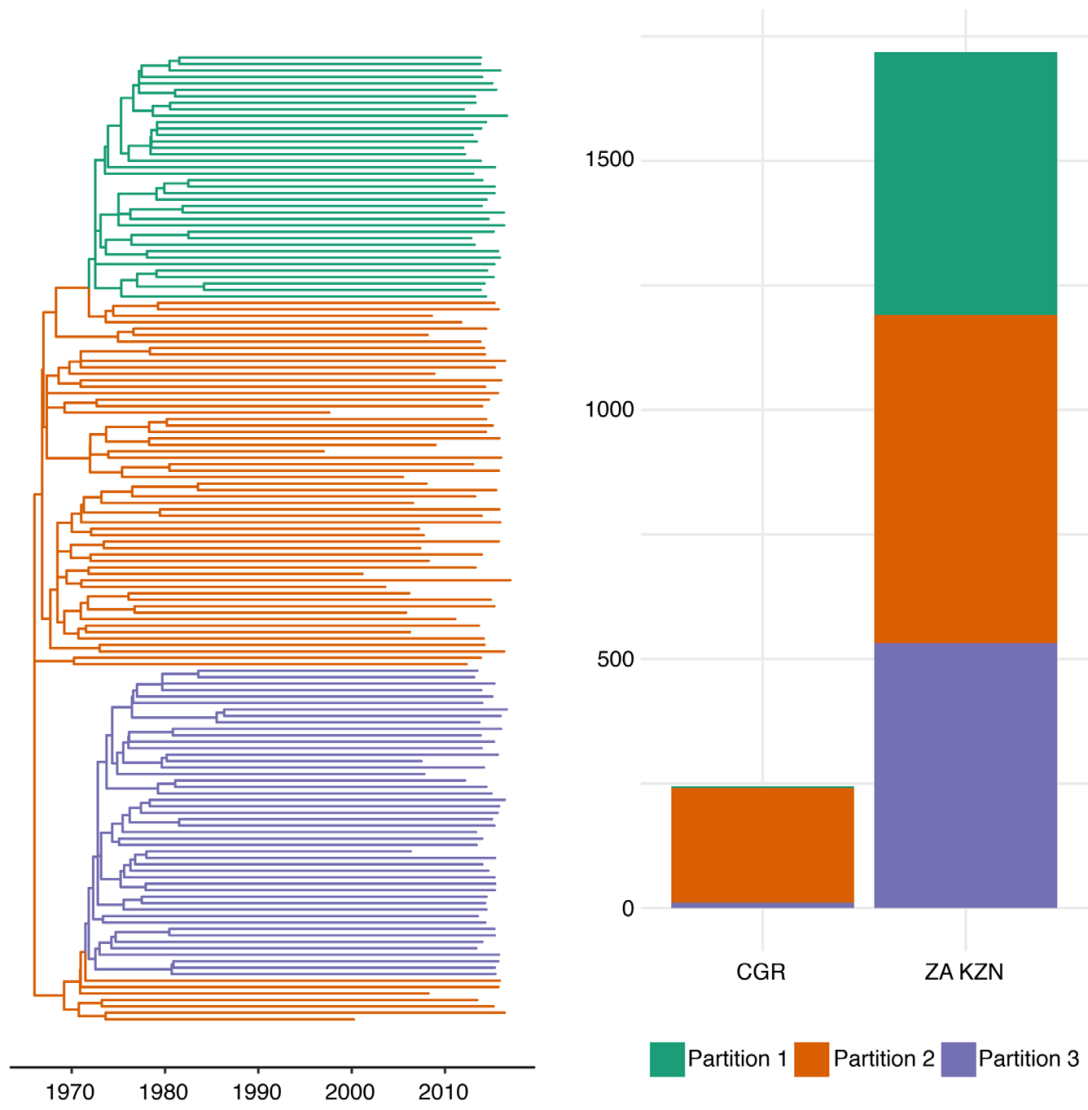
A down sampled time-scaled phylogeny based on *gag* sequences from the Hlabisa, South Africa data set. Colours of branches correspond to partitions found with *treestructure*. The histogram on the right shows the distribution of clade membership among CGR and South Africa (ZA KZN: Hlabisa, KwaZulu-Natal) sequences. Most CGR sequences appear in a single partition.

We observed evidence that the process of migration and lineage importation was not evenly distributed through the population. The vast majority of lineage importation events were associated with a single partition within the HIV phylogeny. Figure 5 shows a down sampled time-scaled phylogeny for the *gag* gene and the distribution of CGRs in different partitions detected using *treestructure* (see Methods). In the *gag* phylogeny, 230 of 244 CGRs clustered within a single partition. In the *pol* phylogeny, 199 of 205 CGRs clustered within a single partition. Finally, in the *env* phylogeny, 174 of 192 CGRs clustered within a single partition.

## Discussion

Our data set enabled precise estimates of early epidemic growth rates, and model-based estimates were, in general, in agreement with non-parametric estimates for early growth rates ( Figure 3 and Figure 4).

The Hlabisa HIV treatment and care programme initially had 1,800 PLWHIV enrolled and receiving ART in 2006, and was rapidly scaled up so that by the end of 2008, 7,576 PLWHIV were enrolled and receiving ART ( Houlihan *et al.*, 2011). Our models also showed a steady decline in transmission rates after 2005, coinciding with ART in Hlabisa ( Figure 2).

Vandormael *et al.* (2019), using different epidemiological methods, reported an overall decline of 43% in incidence rate between 2012 and 2017 using data from Hlabisa. They observed that incidence declined after 2012 and 2014 for males and females, respectively, when approximately 35% of opposite-sex individuals were on ART. We simulated up to 2015 and we observed approximately 35% of individuals on ART in 2014. Between 2014 and 2015, we found a reduction in incidence of 52% for *gag* [from 20.54 (CI = 9.44 – 38.81) to 9.85 (CI = 4.29 – 18.81)], 56% for *pol* [from 83.13 (CI = 31.63 – 400.11) to 36.13 (CI = 13.09 to 169.79)] and 54% for *env* [from 30.75 (CI = 9.83 – 130.13) to 13.99 (CI = 3.74 – 60.99)].

Johnson *et al.* (2017), using a mathematical model for the South African HIV epidemic, estimated that incidence for KwaZulu-Natal peaked in 1997–1998. Our model-based analyses showed that for median values, the incidence peaked in 1988 using both *gag* and *env* genes, and in 1992 using the *pol* gene ( Figure 3). Even though our analysis based on *gag* and *env* genes showed a median value much earlier than expected, the 95% credible interval peaked in 1992. Johnson *et al.* (2017) also showed that incidence estimates varied substantially depending on province. For example, incidence in Western Cape peaked in 2003–2004. In this context, it is possible that for Hlabisa, incidence peaked slightly earlier than the rest of KwaZulu-Natal.

### Model limitations

Our model-based phylodynamic analysis did not account for within-host evolution. This is a common but not universal assumption of population genetic modelling of infectious diseases which treats each infected host as a unit that corresponds to a single pathogen lineage. Bias due to neglecting within-host evolution is known to depend on sample proportion ( Volz *et al.*, 2017), and since these proportions were small in our cohort, is likely to be negligible. Our results may depend on variables which were not included in our epidemiological models, such as sex and age structure. It is likely that transmission rates vary between these categories. However, given the tenuousness of conclusions generated with the current models, it is also unlikely that the present data would support estimation of additional parameters for these categories. Finally, accurate estimation of global importation rates depends on inclusion of appropriate samples to represent diversity present in the global HIV reservoir. We have used all available sequences from GenBank and PANGEA for this purpose. However, inclusion of national-level databases could potentially identify additional lineages that are closely related to South African samples. Thus, the true rate of importation from the global reservoir may exceed the rates estimated here.

### Migration and the role of the global HIV reservoir

We estimated the proportion of imports of HIV-1 lineages from outside the region sampled in South Africa. We summarised these estimates using a statistic that represents the probability that a new infection was endogenous, meaning that the new infection arose via transmission between individuals residing in South Africa. Exogenous transmission can arise from a variety of mechanisms ( Rasmussen *et al.*, 2018). For example, an individual may become infected when residing outside South Africa and then change residence to within South Africa. Alternatively, an individual from within South Africa may travel temporarily outside South Africa and become infected. Finally, an individual with primary residence outside South Africa may travel to South Africa and infect a local resident. We cannot distinguish between these three different mechanisms from the available data, as all three have very similar if not identical consequences for the HIV phylogeny.

Using *gag*, *pol* and *env* genes, we estimated a high probability of new infections in Hlabisa arising from immigration or transmission from external sources. The estimates from different genes had overlapping credible intervals.

Our estimated proportion of 62% (95% CI: 40% – 78%) of new infectious imported to Hlabisa using the *pol* gene was higher than the recent report of 35% (95% CI = 20% – 60%) in Rasmussen *et al.* (2018). Rasmussen *et al.* (2018) used a very similar model-based phylodynamic approach which also utilised *pol* sequence data from KwaZulu-Natal, but their catchment excluded the Hlabisa sub-district. We are not able to determine whether differences between our results and Rasmussen *et al.* (2018) were due to true differences between communities, patients sampled, or differences in inference methods, but both analyses support the presence of a very large proportion of transmissions occurring between rather than within communities.

In the phylogenetic tree, we observed a strong association between cladistic structure and placement of CGR sequences. The majority of CGRs were placed within a single partition. Similar arrangements were observed for HIV-1 subtype C in Ethiopia in which distinct clades composed by sequences from a single catchment ( Amogne *et al.*, 2016) grouped together. Both examples in Ethiopia and South Africa (involving a single catchment area) could potentially represent local evolving sub-epidemics. Another possibility is that these arrangements arose from unmeasured heterogeneity in the population, such as if a subset of the population, possibly a high-risk core group, has higher rates of migration outside of the sample catchment. In Hlabisa, males and females migrate to other areas in South Africa to work and males migrate further than females and return home less frequently. In addition, being a migrant and having lived in four or more places were found to be significant risk factors for HIV-1 infections ( Lurie, 2006). Migrants had a 69% increase in acquiring HIV when compared to non-migrants due to increased prevalence of HIV risk behaviours ( Dzomba *et al.*, 2019). As an example of differences in behaviour: men who migrate to Carletonville, which is approximately 700 km away from Hlabisa, return home three to four times a year and the likelihood of infecting their partners is very low ( Lurie, 2006). These behaviours suggest that there exists a subset within Hlabisa that is more likely to be infected by an external source. However, we were unable to test this hypothesis as we would need information on whether a patient was a migrant or not at the time of seroconversion.

## Data availability

### Underlying data

Genetic HIV-1 sequence data used in our analyses are available on request to the PANGEA consortium ( https://www.pangea-hiv.org/). We are currently working on getting permission to release sequence into GenBank.

The R and Python scripts used in our analyses are available as a research compendium in GitHub ( https://github.com/thednainus/pangeaZA).

### Extended data

Analysis code available from: https://github.com/thednainus/pangeaZA

Archived analysis code as at time of publication: https://doi.org/10.5281/zenodo.6532287 ( Nascimento, 2022)

License: GNU-LGPL
