# Supplementary material for: Evaluating whole HIV-1 genome sequence for estimation of incidence and migration in a rural South African community
Source: Wellcome Open Res. 2022 Jun 21;7:174. [Version 1] doi: 10.12688/wellcomeopenres.17891.1 (PMC10276198; doi:10.12688/wellcomeopenres.17891.1)
Supplement: Peer review history

# Peer review: Reviewer response for version 1

**DOI:** 10.21956/wellcomeopenres.19822.r58273

**Published:** 2023-06-16

**Authors:** Stephane Hue

**Affiliations:** 1 Department of Infectious Disease Epidemiology, London School of Hygiene and Tropical Medicine, London, UK

This study conducted in Hlabisa, Kwazulu-Natal, aimed to understand the transmission patterns of HIV-1 subtype C in the area, and to estimate various epidemiological parameters including transmission rates, viral effective population size, the effective number of infections over time and the proportion of infections originating from outside the community. The authors used a very thorough and up to date phylodynamics framework to analyse 2,503 local HIV-1 genetic sequences together with a large set of genetically close global references (CGRs). The results showed similar high trends in epidemic growth rates between 1980 and 1990, followed by a steep decline. Estimates using the *pol* gene tended to be higher than those estimated using the *gag* or *env* genes. Mean estimates of the proportion of new infections originating externally were rather high (between 62% and 85%, depending on the gene region) indicating a significant level of inter-connectedness between communities in rural South Africa.

 Most of the objectives of this analysis were aligned with that of previous work done by Rasmussen *et al.* and/or Wilkinson *et al.,* sometimes yielding different results. One key difference with previous analyses is the addition of non-African sequences to the dataset, expanding the scope of the migration analysis, and a comparison of estimates obtained from different HIV-1 genomic regions. The extent to which these additions generate new knowledge could be debated but the work is sufficiently novel and expertly conducted to be of interest to the HIV research community.

 The phylogenetic and phylodynamic analyses are very well designed and conducted. The methodology is sound, appropriate and appreciably detailed in the manuscript. The authors have made appreciable efforts to make their data and scripts  easily accessible, except for the sequence data which is only available on request. Note that I cannot comment on the epidemiological models, as modelling lies outside of my expertise.

 The results seem robust and adequately interpreted. If anything, I find the reporting of the lineage importation analysis a bit too general. It is mostly circumscribed to estimates of the probability that a new infection was not attributable to endogenous transmission, but given that these probabilities are rather large, it would have been useful to describe the close global references related to these exogenous transmissions a bit more. As it stands, we don’t know much about where these CGRs come, whether particular sampling locations are more likely to be associated with import into Hlabisa etc.

 The discussion is thorough and well documented, with insightful efforts to interpret differences with the previous work of Rasmussen *et al*. I do find the high proportion of imported new infections rather intriguing, but not concerning (concerning for the publication; it is of course concerning for the population at risk in Hlabisa…).

 Minor comments:

1. The choice of some models (e.g. the HKY substitution model, the uncorrelated lognormal relaxed clock model) is not justified. Were model testing procedures used to determine goodness of fit? Are these choices based on previous work? This should be clarified.
2. It is said that the *pol* gene dataset was reduced to match the size of the gag and env ones. How was the down-sampling done?
3. The section on phylogenetic partitioning could be clearer. I understand the concept of partitions with different coalescent time distributions, but I am not sure I understand why partitioning is important here. Maybe the authors could expand a bit on why they partitioned the tree and what these partitions mean in epidemiological terms.

Is the work clearly and accurately presented and does it cite the current literature?

Yes

If applicable, is the statistical analysis and its interpretation appropriate?

I cannot comment. A qualified statistician is required.

Are all the source data underlying the results available to ensure full reproducibility?

Partly

Is the study design appropriate and is the work technically sound?

Yes

Are the conclusions drawn adequately supported by the results?

Yes

Are sufficient details of methods and analysis provided to allow replication by others?

Yes

Reviewer Expertise:

Viral genomics, molecular epidemiology,  phylodynamics

I confirm that I have read this submission and believe that I have an appropriate level of expertise to confirm that it is of an acceptable scientific standard.

# Peer review: Reviewer response for version 1

**DOI:** 10.21956/wellcomeopenres.19822.r56178

**Published:** 2023-05-05

**Authors:** Sebastian Duchene

**Affiliations:** 1 Department of Biochemistry and Molecular Biology, The University of Melbourne, Melbourne, Victoria, Australia

This is an interesting study about the epidemiological dynamics of HIV-1 (subtype C) in a rural South African community. The process of data collection and curation is very thorough and the phylodynamic models are very neatly tailored to the problem of distinguishing importations from local transmission. My only major concern here is that there is not enough detail to follow what the phylodynamic model is doing. I accessed the github repositories, but again, there is not enough information there to understand it. Below I list specific points and suggestions before I would recommend this work for indexing:

Major points:

- Equations (1) model infected individuals that are infectious (_I_), in ART and thus not infectious (_Z_), and the size of the global reservoir (_X_). First, it would be useful to state clearly here that tips in the trees are labeled as belonging to either of these demes. Second, shouldn't there be a migration term here? Due to the way the data set is constructed, the global reservoir consists of singleton sequences, so I can't see how parameters related to _X_ can be estimated without some sort of migration matrix. Moreover, distinguishing importations from local transmission would from a modelling standpoint would also require such a migration parameter.
- I am not clear on the rationale for removing so called 'redundant' non South African sequences. Is this just to make the data set more manageable, or would it have any impact on the model? In particular, wouldn't including such sequences inform about the coalescent rate from the 'reservoir' deme?
- I am aware that some of the authors have presented this in the past, but it would be very useful to include an expression, or at least a short section of text as to how the ODEs are integrated into the coalescent likelihood. As it reads presently, the epi modelling and phylodynamics are a bit disjointed.

Minor comments:

- On the less technical side, I am a bit concerned with the final sentences about migrants being more likely to acquire HIV in these communities. I can see that there is some empirical evidence from the references, but none of this was tested in this study because such metadata on seroconversion is not available. I would suggest making this section a bit less speculative.
- Check that the abbreviation of 'confidence interval (CI)' actually occurs when it is first mentioned. I don't have page numbers, but this is somewhere at the start of the Introduction.
- When discussing the role of importations and the global HIV reservoir, the probabilities are in percentages. Formally, probabilities should be between 0 and 1, to avoid confusion with other quantities (e.g. risk).

Is the work clearly and accurately presented and does it cite the current literature?

Partly

If applicable, is the statistical analysis and its interpretation appropriate?

Yes

Are all the source data underlying the results available to ensure full reproducibility?

Partly

Is the study design appropriate and is the work technically sound?

Yes

Are the conclusions drawn adequately supported by the results?

Yes

Are sufficient details of methods and analysis provided to allow replication by others?

Partly

Reviewer Expertise:

infectious disease phylodynamics, phylogenetics, virus evolution

I confirm that I have read this submission and believe that I have an appropriate level of expertise to confirm that it is of an acceptable scientific standard, however I have significant reservations, as outlined above.

# Peer review: Reviewer response for version 1

**DOI:** 10.21956/wellcomeopenres.19822.r51174

**Published:** 2022-06-27

**Authors:** Samuel Alizon

**Affiliations:** 1 Center for Interdisciplinary Research in Biology (CIRB), College de France, CNRS, INSERM, Université PSL, Paris, France

Summary

 The authors study the HIV epidemic in a rural South-African community by applying phylogenetic and phylodynamic methods to a massive dataset comprising thousands of full genome sequences. They first estimated the temporal dynamics of incidence and prevalence and showed using the phydynR package that the former peaked around 1990. They further investigated these trends using a non-parametric coalescent approach. Finally, they use the phylogenetic structure to analyse migration patterns and show that the majority of the transmission events have their origin outside of the studied community. They analyse these results in the context of the South-African HIV epidemic and discuss potential limitations such as the absence of within-host evolution.

 Review:

 This study applies state-of-the-art inference software packages to a massive dataset to tackle a timely public-health question. My comment has to do with the interpretation of the results, which could perhaps be slightly more exhaustive (especially the comparison between gene regions and between methods). I think the model could also benefit from a few details.

  

1.  Interpretation of the results
  1.1 One of the added values of this study is that the authors perform their analyses on three different regions of the HIV genome. Although their results are qualitatively similar between these regions, there are some noticeable differences. In particular, with the phydynR approach, the epidemic appears to have a larger size when the analyses are performed on POL than when they are performed on GAG (with also a slightly slower epidemic). I think this trend should be discussed further. Is it due to the fact that there are more POL sequences?
  1.2 Similarly, another asset of the approach is that the authors use two phylodynamics methods to infer past epidemic trends ( *phydynR* and *skygrowth*). In the main text, they write that the two methods are consistent but I found this to be perhaps a bit hasty. Indeed, when comparing Figure 3B with Figure 4, there appear to be some differences. For example, for most partitions and genes, the prevalence peaks after 2000, whereas it is not the case in Figure 3B. Again, it would be interesting to discuss the potential origin of these divergences in the manuscript.
  1.3 Intuitively, I would have expected the genomes resulting from introductions that are external to the community to cluster together. However, from the way the manuscript was written, I think my intuition is perhaps wrong. But I would appreciate it if the authors could clarify this point. Also regarding the proportion of transmission from outside the community, I was surprised that these appear to be the rule than the exception and I think it would be interesting to discuss this further (at least for a wide audience).
  1.4 I found that the relatively early epidemic peak was not really discussed. The authors mention the test & treat policy in the Discussion (which might perhaps also deserve to be mentioned in the introduction) but the particular initiative cited here started in 2006, i.e. more than 10 years after the epidemic peak inferred here. I think that giving a few details about the history of the measures implemented to control the HIV epidemic in Hlabisa in the Introduction could help better interpret the results obtained.
2. Methods
  2.1 I found that some features of the model were unclear:
  2.1.1 I was expecting to see a migration term in the equation for $ ***dI/dt*** $ (otherwise, I wonder why to introduce this process at all in the model it). 
  2.1.2 It was unclear to me why to mention $ ***dZ/dt**$ *and $ ***dX/dt*** $since they do not affect the dynamics of $ ***dI/dt*** $(unless a term is missing).
  2.1.3 About $dX/dt$, since this is the equation for exponential growth, wouldn't be simpler to directly write that ***X(t)=X_0 Exp(\rho t***)?
  2.1.4 I found the notation $\ ***alpha(t)*** $ potentially a bit misleading because, if I understand correctly, it corresponds to something like $ ***a \tau*** $, where $ ***a=0*** $ before 2005.
  2.1.5 From what I understood, the value of $\alpha(t)$ is fixed (it is not listed in Table 1). If so, the authors should explain how this value was set and, ideally, perform a sensitivity analysis. 
  2.1.6 Given all these unknowns about $\alpha(t)$, I am not sure I understand the rationale for its introduction because $ ***\beta(t)*** $ will already be capturing the decrease in epidemic growth. 
  2.2 In the Discussion (page 8), the authors indicate that they performed "simulations", which might explain some of the model features I found surprising in 2.1. If this is the case, the simulation results should be presented in the Results section and the methods could be clarified. 
  2.3 Wouldn't it make sense to have the lineage importation parameter vary with time?

**Minor comments**

 (I apologize for the lack of clarity in these comments but since Wellcome Open Research does not bother to add line numbers this is the best I can do)

- page 3: The formulation "We evaluated the potential of [...] methods" seems inaccurate since this work is more about applying methods rather than comparing them or comparing their output to that obtained using other types of data.
- page 3: It was unclear to me whether the genomic data analysed in the context of this study was original (in which case the authors should specify it and it would clearly increase the impact of the study) or if they were re-analysing data that was already published in another context.
- page 4: The proportion of sequences with recombination events seems very low (16 in more than 2400 sequences). Is this me being wrong about the occurrence of these events (which is perhaps the most likely) or does this result from some of the filtering steps performed beforehand (e.g. subtype selection)?
- page 4: With such a massive dataset I would have naively expected a GTR model to fit better than an HKY model. Was there any specific reason for the model choice here?
- page 4: The authors do not seem to discuss the robustness of the phylogenetic signal in the data.
- page 6: It would perhaps be interesting to plot the prior distribution and the posterior for the migration rates.

Is the work clearly and accurately presented and does it cite the current literature?

Yes

If applicable, is the statistical analysis and its interpretation appropriate?

Partly

Are all the source data underlying the results available to ensure full reproducibility?

Partly

Is the study design appropriate and is the work technically sound?

Yes

Are the conclusions drawn adequately supported by the results?

Partly

Are sufficient details of methods and analysis provided to allow replication by others?

Partly

Reviewer Expertise:

epidemiology, evolutionary biology, mathematical modelling

I confirm that I have read this submission and believe that I have an appropriate level of expertise to confirm that it is of an acceptable scientific standard, however I have significant reservations, as outlined above.
